# Effects of duration of long-acting GnRH agonist downregulation on assisted reproductive technology outcomes in patients with adenomyosis: a retrospective cohort study

**DOI:** 10.3389/fmed.2023.1248274

**Published:** 2023-09-26

**Authors:** Yexing Li, Li Ge, Xiaohe Yang, Linlin Cui, Zi-Jiang Chen

**Affiliations:** ^1^Center for Reproductive Medicine, Cheeloo College of Medicine, Shandong University, Jinan, Shandong, China; ^2^Research Unit of Gametogenesis and Health of ART-Offspring, Chinese Academy of Medical Sciences (No. 2021RU001), Jinan, Shandong, China; ^3^Key Laboratory of Reproductive Endocrinology of Ministry of Education, Shandong University, Jinan, Shandong, China; ^4^Shandong Key Laboratory of Reproductive Medicine, Shandong Provincial Hospital Affiliated to Shandong First Medical University, Jinan, China; ^5^Shandong Provincial Clinical Research Center for Reproductive Health, Jinan, Shandong, China; ^6^Shandong Technology Innovation Center for Reproductive Health, Jinan, Shandong, China; ^7^National Research Center for Assisted Reproductive Technology and Reproductive Genetics, Shandong University, Jinan, Shandong, China; ^8^Center for Reproductive Medicine, The Second Hospital of Shandong University, Cheeloo College of Medicine, Shandong University, Jinan, Shandong, China; ^9^Shanghai Key Laboratory for Assisted Reproduction and Reproductive Genetics, Shanghai, China; ^10^Center for Reproductive Medicine, Ren Ji Hospital, School of Medicine, Shanghai Jiao Tong University, Shanghai, China

**Keywords:** adenomyosis, GnRH agonist, downregulation, pregnancy outcome, assisted reproductive technology

## Abstract

**Objectives:**

To elucidate the relationship between long-acting GnRH agonist (GnRHa) downregulation and assisted reproductive technology (ART) outcomes and identify the optimal duration of downregulation in patients with adenomyosis.

**Design:**

Retrospective cohort study.

**Participants:**

The study was designed to evaluate ART outcomes in adenomyosis patients with and without GnRHa downregulation between January 2016 and December 2020. A total of 374 patients with adenomyosis (621 cycles) were included with 281 cycles in downregulation group versus 340 cycles in non-downregulation group. After 1:1 propensity score matching (PSM), a sample size of 272 cycles in each group was matched. The matched downregulation group was further divided into 1-month (147 cycles), 2-months (72 cycles), and ≥3 months downregulation (53 cycles) subgroups. Stratification analysis was conducted on pregnancy outcomes in 239 fresh embryo transfer (ET) cycles and 305 frozen embryo transfer (FET) cycles.

**Results:**

The downregulation group had larger mean diameter of initial uterus and higher proportion of severer dysmenorrhea compared to non-downregulation group. The pregnancy-related parameters in GnRHa downregulation group were similar to those in non-downregulation group, except for higher late miscarriage rate (MR) (13.4% vs. 3.1%, *P* = 0.003). The subgroup comparisons in fresh ET cycles indicated that implantation rate (75.0% vs. 39.2%, *P* = 0.002), biochemical pregnancy rate (91.7% vs. 56.0%, *P* = 0.036) and clinical pregnancy rate (83.3% vs. 47.0%, *P* = 0.016) could be improved by prolonged GnRHa downregulation (≥3 months), whereas late MR was difficult to be reversed (30.0% vs. 3.2%, *P* = 0.017). In FET cycles, higher MR (53.6% vs. 29.9%, *P* = 0.029; 58.8% vs. 29.9%, *P* = 0.026) and lower live birth rate (18.8% vs. 34.1%, *P* = 0.023; 17.1% vs. 34.1%, *P* = 0.037) were observed in the 1-month and ≥3 months downregulation group, while no differences were found in the 2-months downregulation group compared to the non-downregulation group.

**Conclusion:**

In patients with severer adenomyosis, long-acting GnRHa downregulation might be correlated with improved ART outcomes. In fresh ET cycles, prolonged downregulation (≥3 months) might be beneficial to improve live birth rate, which needed to be verified by further study with larger sample. In FET cycles, the optimal duration of downregulation was not certain and still needed further exploration.

## Key points

1. Firstly, we adopted PSM approach to eliminate the influence of confounding factors and enhance the comparability between the downregulation group and non-downregulation group.
2. Secondly, subgroup analysis was performed to evaluate the dose-response relationship between the duration of long-acting GnRHa downregulation and pregnancy outcomes.
3. Thirdly, stratification analysis was used to evaluate the different downregulation strategies in fresh ET and FET cycles.
4. The study was single-center, retrospective, and the sample size was limited.

## Introduction

Adenomyosis, an estrogen-dependent disease, is characterized by the invasion of endometrial gland and stroma into the myometrium, which contributes to uterine enlargement, abnormal uterine bleeding, chronic pelvic pain and infertility (1–3). The performances of adenomyosis by ultrasound and magnetic resonance imaging (MRI) are manifold and elusive (4). In the field of assisted reproductive technology (ART), adenomyosis was a non-negligible factor for patients with infertility, and its prevalence in women ≥ and <40 years old were respectively 29.7% and 22.0% (5). One previous meta-analysis indicated that implantation rate (IR), clinical pregnancy rate (CPR) and live birth rate (LBR) significantly decreased in patients with adenomyosis compared to non-adenomyosis patients receiving *in vitro* fertilization (IVF) treatment (6). Additionally, adenomyosis might increase the occurrence of maternal and neonatal complications, such as preterm birth, preeclampsia, small for gestational age, preterm premature rupture of membranes, postpartum hemorrhage and neonatal intensive care unit admission rate (7–9). The adverse effects of adenomyosis on clinical outcomes might be associated with disruptive endometrial receptivity, aberrant tubal transport, distorted uterine cavity and irregular uterine contractility (10–12).

Long-acting GnRH agonist (GnRHa) is usually used in patients with adenomyosis receiving ART treatment, because it can induce and maintain a hypoestrogenic state by suppressing gonadotropin secretion (13). Moreover, it can induce apoptosis of ectopic endometrium, relieve inflammation reaction and reduce aberrant angiogenesis in adenomyotic lesions, which are beneficial to reduce the uterine size and relieve clinical symptoms (14, 15). Therefore, long-acting GnRHa downregulation was utilized not only before controlled ovarian stimulation (COS) in fresh embryo transfer (ET) cycles but also before endometrial preparation in frozen embryo transfer (FET) cycles. However, there was continuing debating on the effect of long-acting GnRHa downregulation on ART outcomes in patients with adenomyosis. Two retrospective studies elucidated that ultralong GnRHa downregulation protocol improved CPR compared to long and short GnRHa protocols in patients with adenomyosis (16, 17), however, Lan et al.’ (18) study did not support the improvement of CPR. Similarly, in the FET cycles, Niu et al.’ (19) study demonstrated that long-acting GnRHa downregulation followed by hormone replacement therapy (HRT) protocol significantly improved CPR in adenomyosis patients compared with the sole HRT protocol, but no consistent conclusion was supported by another two studies (20, 21). Besides the divergence of the role of long-acting GnRHa downregulation, the duration of downregulation in patients with adenomyosis undergoing ART was also disputable. Previous studies verified that prolonged GnRHa treatment reduced uterine volume and increased uterine elasticity in patients with adenomyosis, which might improve pregnancy outcomes (22–24), whereas the optimum duration of downregulation still needed further exploration.

Consequently, we designed this retrospective study to elucidate the relationship between long-acting GnRHa downregulation and pregnancy outcomes and try to identify the optimal duration of downregulation in patients with adenomyosis undergoing ART treatment.

## Materials and methods

### Study design and population

This retrospective cohort study included patients with adenomyosis who underwent ART at the Center for Reproductive Medicine, Shandong University from January 2016 to December 2020 with the follow-up time of FET up to June 2022. The adenomyosis was diagnosed by transvaginal ultrasound (TVS), which was reviewed independently by two sonographers experienced in gynecologic imaging. The diagnostic standard coincided with the Morphological Uterus Sonographic Assessment (MUSA) statement (25). The typical sonographic features were enlarged uterus with spherical appearance or asymmetrical thickening of myometrium, accompanying with or without echogenic lines and buds under the endometrium, hyperechoic islands, translesional vascularity, fan-shaped shadowing, cystic echogenicity, irregular or interrupted junctional zone (26). Patients with uterine malformation, chromosomal abnormality or preimplantation genetic testing were excluded from the study. The downregulation was defined as the usage of long-acting GnRHa before ET, which might range from 1 to 6 months. Conversely, the absence of long-acting GnRHa was defined as non-downregulation. According to the GnRHa duration, the downregulation group was further divided into 1-month downregulation, 2-months downregulation, and ≥3 months downregulation subgroups.

### Controlled ovarian stimulation

The long-acting GnRHa downregulation was usually used in patients with severer adenomyosis with normal ovarian reserve. The usage of other protocols were decided according to patient’s comprehensive situations. In the ultra-long protocol, patients received the first injection of long-acting GnRHa (Triptorelin Acetate for Injection, 3.75 mg, Ipsen Pharma Biotech, France; Leuprorelin Acetate Microspheres for Injection, 3.75 mg, Lizhu, China; Goserelin Acetate Sustained-Release Depot, 3.6 mg, AstraZeneca, UK) on day 2 or 3 of the menstrual cycle, and the comprehensive evaluation of serum hormone levels, the diameter of follicles and uterine size 28 days later was carried out to determine whether long-acting GnRHa downregulation continued. The standard of pituitary downregulation was defined as low levels of serum luteinizing hormone (LH, < 5 IU/L) and estradiol (E_2_, < 50 pg/ml), thin endometrial thickness (<5 mm), the diameters of follicles <8 mm in bilateral ovary and no existing of functional cysts. After 28 days of the last long-acting GnRHa injection, gonadotropin (Gonal F, Merck Serono, Switzerland; Puregon, MRK, China; Lishenbao, Lizhu, China) 150–300 IU daily was administered for subsequent COS.

The starting doses of gonadotropin were determined by comprehensive consideration of age, body mass index (BMI), ovarian reserve function including parameters of AFC, anti-mullerian hormone (AMH), and serum follicle stimulating hormone (FSH) level. Adjuvant drugs, such as recombinant human lutropin α, metformin and growth hormone, were added according to the principle of individualized treatment. When at least two leading follicles reached 18 mm or greater, 8,000–10,000 IU of urinary human chorionic gonadotropin (hCG, Livzon Pharmaceutical Group. Inc., Zhuhai, China) was administered intramuscularly to trigger oocyte maturation. The dosage and timing of hCG were confirmed basing on the number of dominant follicles, serum E_2_ levels, BMI and ovarian reserve function. Oocyte retrieval guided by TVS was carried out 36–38 h later. Oral dydrogesterone tablet (Duphaston, Abbott Biologicals B.V., Netherlands) 10 mg twice daily and vaginal progesterone gel (Crinone gel, Merck Serono, Switzerland) 90 mg once daily were administered as luteal phase support. IVF or intracytoplasmic sperm injection (ICSI) was selected in the procedure of fertilization depending on sperm quality. Two high-quality cleavage-stage embryos on day 3 or one blastocyst on day 5 after oocyte retrieval were transferred into the uterus under the guidance of abdominal ultrasound. The high-quality embryos were defined as 2PN (pronuclear)-derived embryos with 7–10 cells and scores ≥ 3 on day 3 and ≥ 4BC on day 5 (27). If fresh ET was canceled or the remaining embryos were present, the eligible embryo would be frozen. The whole embryo freezing was recommended, such as varian hyperstimulation syndrome, elevated serum progesterone on hCG trigger day or hydrosalpinx. Other protocols of COS without long-acting GnRHa had been described in previous studies published by our center (28, 29).

### Endometrial preparation in FET cycles

Patients undergoing GnRHa downregulation combined with HRT received the first injection of 3.75 mg long-acting GnRHa on day 2 or 3 of the menstrual cycle. The results of serum hormone levels and sonographic evaluation 28 days later determined the continuation of long-acting GnRHa. About 28 days after last injection of long-acting GnRHa, HRT was performed for endometrial preparation. Oral estradiol valerate (Progynova; Bayer AG, Germany) was administrated in a dose-escalating method, 6 mg/day for the first 5 days and subsequently 8 mg/day for the next 5 days. After 10 days, 8 mg/day might be continued for 3–4 days according to the comprehensive assessment of the endometrial thickness, and serum hormone levels. When endometrial thickness reached at least 7 mm, oral dydrogesterone tablet 20 mg twice daily and vaginal progesterone soft capsules (Utrogestan, Besins, Belgium) 200 mg once daily were used to transform endometrium. One frozen-thawed blastocyst was transferred into the uterus under the guidance of abdominal ultrasound 5–6 days after the addition of progesterone. After ET, progesterone was continued for luteal phase support. Other protocols for endometrial preparation in FET cycles have been described in the previous study (30).

### Observational parameters and outcome variables

Observational parameters included basic characteristics of patients, the mean diameter of initial uterus, the history of dysmenorrhea and IVF-related parameters. The mean diameter of initial uterus was calculated as the average of long and wide diameters in longitudinal section by TVS between day 2 and day 5 of the menstrual cycle. The dysmenorrhea was classified into none, mild, moderate and severe degrees according to the verbal multidimensional scoring system, which mainly depended on subjective feelings of patients (31). The primary pregnancy outcome was LBR, and the secondary pregnancy outcomes included CPR, IR, biochemical pregnancy rate (BPR), miscarriage rate (MR), early MR and late MR. The definitions of these outcomes based on the International Glossary on Infertility and Fertility Care (32). The serum β-hCG level was examined 14 days after ET. Biochemical pregnancy was defined as a serum β-hCG level ≥ 10 IU/L. Clinical pregnancy was defined as the presence of one or more gestational sacs confirmed by TVS including ectopic pregnancy. Miscarriage was defined as pregnancy loss <28 gestational weeks. Early miscarriage occurred <12 gestational weeks, and late miscarriage occurred ≥12 gestational weeks. Live birth was defined as delivery of at least one viable infant ≥28 gestational weeks. IR was defined as the ratio of total numbers of gestational sac confirmed by TVS to total numbers of transferred embryos.

### Statistical analysis

A 1:1 propensity score matching (PSM) approach using nearest neighbor matching (caliper value = 0.03) was applied to deal with the confounding factors and enhance comparability between groups on R software using “MatchIt” package. Matching variables included age, BMI, duration of infertility, basal FSH, AFC, AMH, the proportion of primary infertility, endometriosis and previous gynecological surgery. Further subgroup analysis was performed using the PSM dataset, which compared pregnancy outcomes of 1-month, 2-months, and ≥3 months downregulation subgroups in the non-downregulation group. Stratification analysis was conducted on pregnancy outcomes in fresh ET and FET cycles. Shapiro–Wilk test was utilized to test the normality of continuous variables. Median (25th–75th percentile) was utilized to describe non-normality distribution variables. Frequencies (percentages) were utilized to describe categorical variables. Continuous variables with non-normality distribution were compared between groups by the Mann–Whitney U test. Categorical variables were compared between groups by χ^2^ test or Fisher’s exact test. Bonferroni method was used to carry out subgroups comparisons. *P* < 0.05 was considered as statistically significant. All statistical analysis were conducted by SPSS 25.0 (SPSS, Inc., Chicago, IL, USA) and R software 4.2.0.

## Results

**FIGURE 1:**
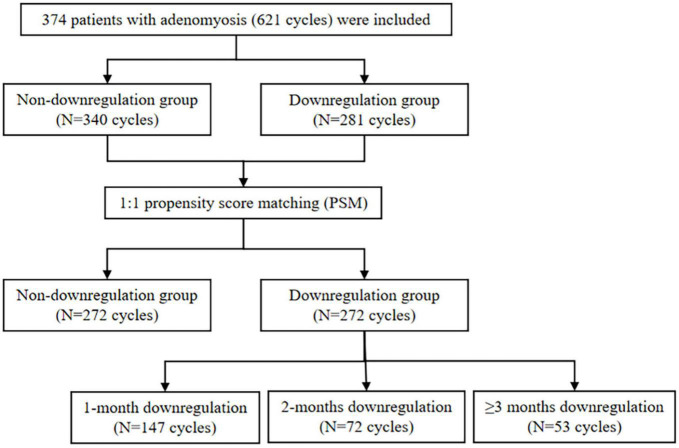
The study population flowchart.

A total of 374 patients with adenomyosis (621 cycles) were included with 281 cycles in downregulation group versus 340 cycles in non-downregulation group. PSM was used to balance the demographical bias between two groups. The flowchart of the study was shown in Figure 1. The characteristics of the study population before and after PSM were shown in Table 1. Before PSM, the downregulation group had younger age, longer duration of infertility, higher proportion of primary infertility and lower basal FSH compared to the non-downregulation group. After PSM, these factors were well balanced. Ultimately it involved a matched sample size with 272 cycles in each group. Except for these matching factors, the downregulation group had larger mean diameter of initial uterus (6.32 vs. 5.50 cm, *P* < 0.001) and higher proportion of severe dysmenorrhea (36.03% vs. 17.28%, *P* < 0.001) compared to the non-downregulation group. The pregnancy outcomes of two groups after PSM were shown in Table 2. No differences were found in the IR, BPR, CPR, MR, early MR, and LBR, while late MR in the downregulation group was significantly higher than the non-downregulation group (13.4% vs. 3.1%, *P* = 0.003).

**TABLE 1:** Characteristics of the study population before and after PSM.

	Before PSM		*P*-value	After PSM		*P*-value
	**Non-downregulation group**	**Downregulation group**		**Non-downregulation group**	**Downregulation group**	
No. of cycles	340	281		272	272	
Age, years	35.00 (31.00, 39.00)	33.00 (31.00, 37.00)	**0.003**	34.00 (30.00, 38.00)	33.50 (31.00, 37.00)	0.537
BMI, kg/m^2^	23.70 (21.64, 26.49)	24.52 (21.88, 27.04)	0.125	24.00 (21.77, 26.82)	24.55 (21.96, 27.01)	0.400
Duration of infertility, years	3.00 (1.50, 4.50)	3.00 (2.00, 5.00)	**0.047**	3.00 (2.00, 4.50)	3.00 (2.00, 5.00)	0.173
Primary infertility, *n* (%)	109 (32.06)	125 (44.48)	**0.001**	105 (38.60)	119 (43.75)	0.223
Endometriosis, *n* (%)	68 (20.00)	61 (21.71)	0.601	54 (19.85)	58 (21.32)	0.672
Previous gynecological surgery, *n* (%)	249 (73.24)	193 (68.68)	0.213	191 (70.22)	189 (69.49)	0.852
Basal FSH, IU/L	6.70 (5.66, 8.19)	6.42 (5.27, 7.67)	**0.008**	6.54 (5.63, 7.66)	6.46 (5.30, 7.67)	0.443
AFC	11.00 (8.00, 16.00)	11.00 (7.00, 16.00)	0.890	12.00 (8.00, 16.00)	11.00 (7.00, 16.25)	0.601
AMH, ng/ml	2.055 (1.045, 3.996)	2.200 (1.162, 4.200)	0.320	2.360 (1.205, 4.274)	2.200 (1.160, 4.200)	0.778
Mean diameter of initial uterus, cm	5.50 (4.70, 6.35)	6.30 (5.55, 7.30)	**<0.001**	5.50 (4.70, 6.35)	6.32 (5.55, 7.31)	**<0.001**
History of dysmenorrhea			**<0.001**			**<0.001**
None, *n* (%)	110 (32.35)	34 (12.10)		79 (29.04)	34 (12.50)	
Mild, *n* (%)	120 (35.29)	73 (25.98)		99 (36.40)	68 (25.00)	
Moderate, *n* (%)	58 (17.06)	75 (26.69)		47 (17.28)	72 (26.47)	
Severe, *n* (%)	52 (15.29)	99 (35.23)		47 (17.28)	98 (36.03)	
Total dosage of Gn, IU	1875.00 (1350.00, 2700.00)	2475.00 (2025.00, 3375.00)	**<0.001**	1912.50 (1425.00, 2700.00)	2475.00 (2025.00, 3375.00)	**<0.001**
Duration of Gn stimulation, days	9.00 (8.00, 11.00)	11.00 (9.00, 12.00)	**<0.001**	9.00 (8.00, 11.00)	11.00 (9.00, 12.00)	**<0.001**
LH on HCG trigger day, IU/L	2.63 (1.61, 5.10)	1.27 (0.77, 2.36)	**<0.001**	2.55 (1.50, 4.98)	1.27 (0.78, 2.38)	**<0.001**
E_2_ on HCG trigger day, pg/ml	2261.00 (1478.25, 3350.50)	2355.00 (1494.00, 3809.00)	0.235	2424.00 (1541.25, 3457.00)	2389.00 (1494.75, 3747.75)	0.713
P on HCG trigger day, ng/ml	0.65 (0.41, 0.93)	0.69 (0.47, 0.98)	0.068	0.68 (0.41, 0.94)	0.69 (0.46, 0.98)	0.281
Endometrial thickness on HCG trigger day, cm	0.95 (0.80, 1.10)	0.95 (0.80, 1.10)	0.641	0.95 (0.80, 1.10)	0.95 (0.80, 1.10)	0.767
No. of oocytes retrieved	8.00 (4.00, 12.00)	9.00 (5.00, 13.00)	**0.034**	9.00 (4.00, 12.25)	9.00 (5.00, 13.00)	0.185
No. of 2PN zygotes retrieved	5.00 (3.00, 8.25)	6.00 (3.00, 9.00)	**0.030**	5.00 (3.00, 9.00)	6.00 (3.00, 9.00)	0.083
No. of high-quality embryos retrieved on Day 3	3.00 (1.00, 5.00)	3.00 (1.00, 5.00)	0.699	3.00 (1.00, 5.00)	3.00 (1.00, 5.00)	0.754

Data were presented as median (25th–75th percentile) for non-normality distribution variables and frequencies (percentages) for categorical variables.

PSM, propensity score matching; BMI, body mass index; FSH, follicle stimulating hormone; AFC, antral follicle count; AMH, anti-müllerian hormone; LH, luteinizing hormone; E2, estradiol; P, progesterone; Gn, gonadotropin; HCG, human chorionic gonadotropin; PN, pronuclear. The meaning of the bold values was that *P* < 0.05.

**TABLE 2:** Comparison of pregnancy outcomes between two groups after PSM.

	Non-downregulation group	Downregulation group	*P*-value
**Pregnancy outcomes,% (*n*/N)**			
IR	43.2 (152/352)	44.7 (152/340)	0.686
BPR	56.3 (153/272)	57.7 (157/272)	0.729
CPR	47.8 (130/272)	46.7 (127/272)	0.797
MR	30.0 (39/130)	40.9 (52/127)	0.067
Early MR	26.9 (35/130)	27.6 (35/127)	0.909
Late MR	3.1 (4/130)	13.4 (17/127)	**0.003**
LBR	33.5 (91/272)	27.6 (75/272)	0.136

PSM, propensity score matching; IR, implantation rate; BPR, biochemical pregnancy rate; CPR, clinical pregnancy rate; MR, miscarriage rate; LBR, live birth rate. The meaning of the bold values was that *P* < 0.05.

In order to investigate the optimal duration of downregulation, we further divided the matched downregulation group into 1-month downregulation (147 cycles), 2-months downregulation (72 cycles) and ≥3 months downregulation (53 cycles) subgroups according to the duration of long-acting GnRHa downregulation. The characteristics of the study population of each subgroup after PSM were displayed in Table 3. Compared to non-downregulation group, the ≥3 months downregulation group had lower basal FSH (6.03 vs. 6.54 IU/L, *P* = 0.008), higher AFC (14.00 vs. 12.00, *P* = 0.027), higher AMH (3.240 vs. 2.360 ng/ml, *P* = 0.049), more numbers of retrieved oocytes (11.00 vs. 9.00, *P* = 0.033) and 2PN zygotes (8.00 vs. 5.00, *P* = 0.002). The proportions of severe dysmenorrhea in the 1-month, 2-months, and ≥3 months downregulation group were respectively 28.57%, 37.50%, and 54.72%, which were obviously higher than the control group (17.28%, *P* < 0.001). The mean diameter of initial uterus in the 1-month (6.10 vs. 5.50 cm, *P* < 0.001), 2-months (6.55 vs. 5.50 cm, *P* < 0.001) and ≥3 months downregulation group (7.20 vs. 5.50 cm, *P* < 0.001) were significantly larger than the non-downregulation group, and it significantly increased in the ≥3 months downregulation group compared to the 1-month downregulation group. The total dosage of gonadotropin in the 1-month (2700.00 vs. 1912.50, *P* < 0.001), 2-months (2250.00 vs. 1912.50, *P* < 0.001) and ≥3 months downregulation group (2250.00 vs. 1912.50, *P* < 0.001) was significantly higher than the non-downregulation group. The duration of gonadotropin in the 1-month (11.00 vs. 9.00, *P* < 0.001), 2-months (10.00 vs. 9.00, *P* = 0.002), and ≥3 months downregulation group (11.00 vs. 9.00, *P* = 0.001) was significantly longer than the non-downregulation group. Likewise, the LH on HCG trigger day in the 1-month (1.23 vs. 2.55, *P* < 0.001), 2-months (1.44 vs. 2.55, *P* < 0.001) and ≥3 months downregulation group (1.11 vs. 2.55, *P* < 0.001) was significantly lower than the non-downregulation group, respectively. No statistical differences were detected in other parameters between downregulation subgroup and the non-downregulation group.

**TABLE 3:** Characteristics of the study population of each subgroup after PSM.

	Non-downregulation group	1-month downregulation	*P*-value	2-months downregulation	*P*-value	≥ 3 months downregulation	*P*-value
No. of cycles	272	147		72		53	
Age, years	34.00 (30.00, 38.00)	34.00 (31.00, 37.00)	0.563	34.50 (31.00, 38.00)	0.718	33.00 (30.00, 36.00)	0.252
BMI, kg/m^2^	24.00 (21.77, 26.82)	24.48 (21.84, 27.21)	0.477	24.05 (21.51, 26.49)	0.864	25.24 (23.22, 26.14)	0.185
Duration of infertility, years	3.00 (2.00, 4.50)	3.00 (2.00, 5.00)	0.223	3.00 (2.00, 5.00)	0.334	3.00 (2.00, 4.50)	0.596
Primary infertility, *n* (%)	105 (38.60)	59 (40.14)	0.759	35 (48.61)	0.124	25 (47.17)	0.244
Endometriosis, *n* (%)	54 (19.85)	27 (18.37)	0.713	20 (27.78)	0.146	11 (20.75)	0.881
Previous gynecological surgery, *n* (%)	191 (70.22)	104 (70.75)	0.910	51 (70.83)	0.919	34 (64.15)	0.381
Basal FSH, IU/L	6.54 (5.63, 7.66)	6.44 (5.26, 7.80)	0.696	6.72 (5.62, 8.44)	0.343	6.03 (5.24, 6.84)	**0.008**
AFC	12.00 (8.00, 16.00)	11.00 (7.00, 14.00)	0.134	11.00 (7.00, 15.25)	0.444	14.00 (10.00, 21.00)	**0.027**
AMH, ng/ml	2.360 (1.205, 4.274)	1.898 (1.056, 3.763)	0.255	2.077 (1.027, 3.783)	0.591	3.240 (1.500, 4.357)	**0.049**
Mean diameter of initial uterus, cm	5.50 (4.70, 6.35)	6.10 (5.30, 7.15)^a^	**<0.001**	6.55 (5.60, 7.09)	**<0.001**	7.20 (6.20, 8.45)^a^	**<0.001**
History of dysmenorrhea			**<0.001**		**<0.001**		**<0.001**
None, *n* (%)	79 (29.04)	22 (14.97)		5 (6.94)		7 (13.21)	
Mild, *n* (%)	99 (36.40)	46 (31.29)		18 (25.00)		4 (7.55)	
Moderate, *n* (%)	47 (17.28)	37 (25.17)		22 (30.56)		13 (24.53)	
Severe, *n* (%)	47 (17.28)	42 (28.57)		27 (37.50)		29 (54.72)	
Total dosage of Gn, IU	1912.50 (1425.00, 2700.00)	2700.00 (2075.00, 3750.00)	**<0.001**	2250.00 (1931.25, 3225.00)	**<0.001**	2250.00 (2025.00, 3225.00)	**<0.001**
Duration of Gn stimulation, days	9.00 (8.00, 11.00)	11.00 (10.00, 12.50)	**<0.001**	10.00 (9.00, 12.00)	**0.002**	11.00 (9.00, 12.00)	**0.001**
LH on HCG trigger day, IU/L	2.55 (1.50, 4.98)	1.23 (0.70, 2.48)	**<0.001**	1.44 (1.02, 2.25)	**<0.001**	1.11 (0.77, 1.84)	**<0.001**
E_2_ on HCG trigger day, pg/ml	2424.00 (1541.25, 3457.00)	2432.00 (1347.00, 3637.50)	0.943	2031.50 (1434.75, 4049.25)	0.696	2742.00 (1986.00, 3809.00)	0.092
P on HCG trigger day, ng/ml	0.68 (0.41, 0.94)	0.73 (0.50, 0.98)	0.141	0.62 (0.41, 0.88)	0.412	0.79 (0.50, 1.05)	0.174
Endometrial thickness on HCG trigger day, cm	0.95 (0.80, 1.10)	1.00 (0.85, 1.10)	0.162	0.90 (0.80, 1.00)	0.330	0.90 (0.80, 1.00)	0.541
No. of oocytes retrieved	9.00 (4.00, 12.25)	9.00 (5.00, 13.00)	0.423	8.00 (5.00, 12.00)	0.842	11.00 (6.00, 14.00)	**0.033**
No. of 2PN zygotes retrieved	5.00 (3.00, 9.00)	5.00 (3.00, 9.00)	0.404	5.00 (3.00, 8.25)	0.709	8.00 (4.00, 10.00)	**0.002**
No. of high-quality embryos retrieved on Day 3	3.00 (1.00, 5.00)	3.00 (1.00, 5.00)	0.565	3.00 (1.00, 5.00)	0.546	4.00 (2.00, 5.00)	0.372

Data were presented as median (25th–75th percentile) for non-normality distribution variables and frequencies (percentages) for categorical variables. PSM, propensity score matching; BMI, body mass index; FSH, follicle stimulating hormone; AFC, antral follicle count; AMH, anti-müllerian hormone; LH, luteinizing hormone; E2, estradiol; P, progesterone; Gn, gonadotropin; HCG, human chorionic gonadotropin; PN, pronuclear.

^a^1-month downregulation vs. ≥3 months downregulation: statistically significant difference. The meaning of the bold values was that *P* < 0.05.

The comparisons of pregnancy outcomes among subgroups were shown in Table 4. There were no significant differences in IR, BPR, CPR, early MR, and LBR in the 1-month, 2-months, and ≥3 months downregulation group compared to the non-downregulation group. However, late MR (11.4% vs. 3.1%, *P* = 0.039) in the 1-month downregulation group significantly increased compared to the non-downregulation group. MR (51.9% vs. 30.0%, *P* = 0.029), especially late MR (22.2% vs. 3.1%, *P* = 0.001) in the ≥3 months downregulation group significantly increased compared to the non-downregulation group. No differences in pregnancy-related parameters were detected in comparisons of downregulation subgroups.

**TABLE 4:** Comparison of pregnancy outcomes and subgroup analysis.

	Non-downregulation group	1-month downregulation	*P*-value	2-months downregulation	*P*-value	≥ 3 months downregulation	*P*-value	*P*_*adjust*_
**Pregnancy outcomes,% (*n*/N)**								
IR	43.2 (152/352)	44.0 (85/193)	0.846	41.0 (34/83)	0.713	51.6 (33/64)	0.215	0.423
BPR	56.3 (153/272)	60.5 (89/147)	0.396	50.0 (36/72)	0.343	60.4 (32/53)	0.579	0.302
CPR	47.8 (130/272)	47.6 (70/147)	0.973	41.7 (30/72)	0.354	50.9 (27/53)	0.675	0.558
MR	30.0 (39/130)	38.6 (27/70)	0.219	36.7 (11/30)	0.478	51.9 (14/27)	**0.029**	0.423
Early MR	26.9 (35/130)	27.1 (19/70)	0.973	26.7 (8/30)	0.977	29.6 (8/27)	0.774	0.963
Late MR	3.1 (4/130)	11.4 (8/70)	**0.039**	10.0 (3/30)	0.240	22.2 (6/27)	**0.001**	0.314
LBR	33.5 (91/272)	29.3 (43/147)	0.379	26.4 (19/72)	0.253	24.5 (13/53)	0.202	0.777

IR, implantation rate; BPR, biochemical pregnancy rate; CPR, clinical pregnancy rate; MR, miscarriage rate; LBR, live birth rate. *P_adjust_*: Adjust *p*-values using Bonferroni method in comparison of three subgroups. The meaning of the bold values was that *P* < 0.05.

The comparison of pregnancy outcomes stratified by fresh ET or FET cycles were shown in Table 5. A total of 239 fresh ET cycles and 305 FET cycles were included in the stratification analysis. In fresh ET cycles, IR (75.0% vs. 39.2%, *P* = 0.002), BPR (91.7% vs. 56.0%, *P* = 0.036), CPR (83.3% vs. 47.0%, *P* = 0.016) and late MR (30.0% vs. 3.2%, *P* = 0.017) in the ≥3 months downregulation group significantly increased compared to the non-downregulation group, while no differences were found in MR, early MR and LBR. In intra-subgroup analysis, IR (75.0% vs. 45.5%, *P* = 0.046) in the ≥3 months downregulation group significantly increased compared to the 1-month downregulation group. In FET cycles, MR (53.6% vs. 29.9%, *P* = 0.029; 58.8% vs. 29.9%, *P* = 0.026) significantly increased and LBR (18.8% vs. 34.1%, *P* = 0.023; 17.1% vs. 34.1%, *P* = 0.037) significantly decreased in the 1-month and ≥3 months downregulation group compared to the non-downregulation group. However, no differences in pregnancy outcomes were found in the 2-months downregulation group compared to the non-downregulation group.

**TABLE 5:** Comparison of pregnancy outcomes stratified by fresh ET or FET cycles and subgroup analyses.

	Non-downregulation group	1-month downregulation	*P*-value	2-months downregulation	*P*-value	≥ 3 months downregulation	*P*-value	*P*_*adjust*_
**Fresh ET, % (*n*/N)**								
IR	39.2 (82/209)	45.5 (56/123)^a^	0.261	54.2 (13/24)	0.159	75.0 (15/20)^a^	**0.002**	**0.046**
BPR	56.0 (75/134)	64.1 (50/78)	0.246	66.7 (10/15)	0.427	91.7 (11/12)	**0.036**	0.165
CPR	47.0 (63/134)	53.8 (42/78)	0.337	60.0 (9/15)	0.340	83.3 (10/12)	**0.016**	0.154
MR	30.2 (19/63)	28.6 (12/42)	0.861	33.3 (3/9)	1.000	40.0 (4/10)	0.798	0.769
Early MR	27.0 (17/63)	16.7 (7/42)	0.217	22.2 (2/9)	1.000	10.0 (1/10)	0.446	0.761
Late MR	3.2 (2/63)	11.9 (5/42)	0.175	11.1 (1/9)	0.334	30.0 (3/10)	**0.017**	0.294
LBR	32.8 (44/134)	38.5 (30/78)	0.407	40.0 (6/15)	0.577	50.0 (6/12)	0.377	0.749
**FET, % (*n*/N)**								
IR	49.0 (70/143)	41.4 (29/70)	0.301	35.6 (21/59)	0.083	40.9 (18/44)	0.350	0.771
BPR	56.5 (78/138)	56.5 (39/69)	1.000	45.6 (26/57)	0.165	51.2 (21/41)	0.549	0.475
CPR	48.6 (67/138)	40.6 (28/69)	0.278	36.8 (21/57)	0.135	41.5 (17/41)	0.425	0.874
MR	29.9 (20/67)	53.6 (15/28)	**0.029**	38.1 (8/21)	0.479	58.8 (10/17)	**0.026**	0.394
Early MR	26.9 (18/67)	42.9 (12/28)	0.126	28.6 (6/21)	0.878	41.2 (7/17)	0.249	0.564
Late MR	3.0 (2/67)	10.7 (3/28)	0.301	9.5 (2/21)	0.240	17.6 (3/17)	0.088	0.795
LBR	34.1 (47/138)	18.8 (13/69)	**0.023**	22.8 (13/57)	0.122	17.1 (7/41)	**0.037**	0.757

ET, embryo difference. transfer; FET, frozen embryo transfer; IR, implantation rate; BPR, biochemical pregnancy rate; CPR, clinical pregnancy rate; MR, miscarriage rate; LBR, live birth rate. *P_adjust_*: Adjust *p*-values using Bonferroni method in comparison of three subgroups.

^a^1-monthdownregulation vs. ≥3 months downregulation: statistically significant. The meaning of the bold values was that *P* < 0.05.

## Discussion

In our study, an important demographic characteristic was that symptoms of adenomyosis in the downregulation subgroups were severer than those in the non-downregulation group. For adenomyosis patients with severer disease status, long-acting GnRHa downregulation during ART treatment might be related to improved pregnancy outcomes, which needed to be further verified by larger sample studies. In fresh ET cycles, prolonged downregulation (≥3 months) could significantly improve IR, BPR and CPR, while late MR was not synchronously improved. In FET cycles, the optimal duration of downregulation still needs further exploration.

Adenomyosis is a complex disease with eight classical imaging characteristics. Previous studies reported that the number of sonographic features of adenomyosis might be a prognostic indicator of the severe extent of adenomyosis. Patients with more sonographic features were inclined to have larger uterus and severer symptoms, which were intimated with adverse pregnancy outcomes (33–36). In our study, larger mean diameter of initial uterus and higher proportion of severe dysmenorrhea were observed in the downregulation group. However, IR, BPR, CPR, MR, early MR, and LBR were similar between the downregulation group and non-downregulation group, which suggested that long-acting GnRHa downregulation during ART treatment might be related to improved pregnancy outcomes in patients with severer adenomyosis. Additionally, a recent meta-analysis concluded similar conclusions, that was, patients with adenomyosis receiving short GnRHa downregulation protocol were related to significantly decreased CPR and increased MR, whereas the similar correlation was not found in ultra-long GnRHa protocol (37).

In fresh ET cycles, we noticed that IR, BPR, CPR and MR in the 1-month and 2-months downregulation groups were similar to those in the non-downregulation group. After prolonging downregulation (≥3 months), IR, BPR and CPR significantly improved compared to the non-downregulation group, which suggested that for patients with severer adenomyosis, prolonged duration of long-acting GnRHa downregulation prior to COS had advantageous impacts on pregnancy outcomes. The improvement of pregnancy outcomes might be associated with the potential effects of GnRHa, such as increased pulsatility index of uterine arteries, improved endometrial blood flow and ameliorated the endometrial receptivity (23, 38, 39). Besides these, Guo et al. (40) elucidated that GnRHa could significantly increase the expression levels of Hoxa10, Hoxa11, Lif, integrin b3 protein and pinopodes during the implantation window, which improved the endometrial receptivity and pregnancy outcomes in mouse model. Apart from pregnancy-related parameters, we also noticed that numbers of obtained oocytes and 2PN zygotes in the ≥3 months downregulation group were significantly higher than the non-downregulation group. Although long-acting downregulation might result in deeper inhibition of ovary, prolonged duration of gonadotropin and increased total dosage of gonadotropin might promote the growth of slow-growing follicles and make follicles synchronous development, which was beneficial to the increase of retrieved oocytes and improvement of the quality of oocytes and embryos. Different from the FET cycles, the COS in fresh ET cycles could cause obvious hyperestrogenic status to a certain extent, which could promote the enlargement of uterus and offset partial effect of previous long-acting GnRHa downregulation. Consequently, the selection of long-acting GnRHa downregulation and its duration should be considered according to the patient’s comprehensive situation, and our findings required further verification.

In FET cycles, for patients with severer adenomyosis, IR, BPR, CPR, early MR and late MR in the downregulation subgroups had no statistical differences with the non-downregulation group. Compared to the non-downregulation group, MR increased and LBR decreased significantly in the 1-month downregulation group, while no differences were observed in the 2-months downregulation group. The optimal duration of downregulation still needs further exploration. Li et al. (24) reported that the uterine volume reduced to below 98.81 cm^3^ before FET could significantly decrease MR and increase LBR in patients with adenomyosis. Additionally, Zhang et al. (41) also proposed that in adenomyosis patients with uterine volume between 56 and 100 cm^3^, better pregnancy outcomes with lower MR and higher LBR could be achieved after long-acting GnRHa downregulation in FET cycles. However, MR in the ≥3 months downregulation group was higher than the non-downregulation group, which might be associated with severer adenomyosis. The increased MR might be associated with defect process of spiral arteries remodeling in the uterine junctional zone and subsequent deep placentation impairment (42, 43). Epithelial-mesenchymal transition (EMT) and fibroblast-to-myofibroblast transdifferentiation (FMT) also played important roles on the continuing development of adenomyosis, which could deteriorate fibrosis of adenomyotic lesions and subsequently weaken the effect of long-acting GnRHa (44, 45). These might be important reasons of negative obstetrical outcomes in patients with adenomyosis, such as increased pregnancy loss and preterm birth, while more detailed mechanisms still needed to be elucidated (46–49). Therefore, long-acting GnRHa downregulation did have positive effects on severer adenomyosis in FET cycles, but the duration of GnRHa still needed further exploration. The factors affecting MR were complex and the effects of GnRHa will gradually vanish with the extension of pregnancy. The detailed mechanism of the correlation between higher MR and adenomyosis needs to be constantly explored.

There were three prominent strengths in our study. Firstly, we adopted PSM approach to eliminate the influence of confounding factors and enhance the comparability between the downregulation group and non-downregulation group. Secondly, subgroup analysis was performed to evaluate the dose-response relationship between the duration of long-acting GnRHa downregulation and pregnancy outcomes. Thirdly, stratification analysis was used to evaluate the different downregulation strategies in fresh ET and FET cycles. Certainly, there were several limitations. The study was single-center, retrospective, and the sample size was limited. The severity of adenomyosis could not be accurately assessed by uterine volume. Therefore, multi-center randomized controlled trials need to be carried out.

## Conclusion

In patients with severer adenomyosis, long-acting GnRHa downregulation might be correlated with improved ART outcomes, which needs to be further investigated by larger sample studies. In fresh ET cycles, prolonged downregulation (≥3 months) might improve live birth. In FET cycles, the optimal duration of downregulation still needs further exploration. Our findings provide some reference for clinicians to make individualized fresh ET and FET protocols for adenomyosis patients with different disease status. The mechanism of the correlation between higher MR and adenomyosis needs constant exploration. Randomized controlled trials with larger samples should be designed to verify our findings in the future.

## Data availability statement

The original contributions presented in this study are included in the article/supplementary material, further inquiries can be directed to the corresponding author.

## Ethics statement

The studies involving human participants were reviewed and approved by the Ethics Committee at the Center for Reproductive Medicine, Shandong University (No. 2021-133). Written informed consent was not required because this is a retrospective study.

## Author contributions

LC designed the study and applied for data. YL and XY collected and analyzed the data. YL and LG drafted the manuscript. LG, LC and Z-JC critically reviewed the manuscript. All authors contributed to the manuscript and approved the submitted version.
